# Use of botulinum toxin as a non-surgical treatment option for idiopathic hallux varus: a case report

**DOI:** 10.1093/jscr/rjad423

**Published:** 2023-07-31

**Authors:** Caleb Gottlich, Alexandria Murphy, Neil Jain, Jerry Grimes

**Affiliations:** Department of Orthopedic Surgery, Texas Tech University Health Sciences Center Lubbock, TX, USA; Department of Orthopedic Surgery, Texas Tech University Health Sciences Center Lubbock, TX, USA; Department of Orthopedic Surgery, Texas Tech University Health Sciences Center Lubbock, TX, USA; Department of Orthopedic Surgery, Texas Tech University Health Sciences Center Lubbock, TX, USA

## Abstract

Hallux varus presents with midline deviation of the hallux at the first metatarsophalangeal joint. If left untreated, it may lead to pain and difficulty with normal daily activities. Here, we present a case of spontaneous hallux varus in an 84-year-old female treated non-operatively with injection of botulinum toxin in the Abductor Hallucis Brevis. Ultrasound guidance with electromyography was used to assist in all injections. Near total symptomatic relief and resumption of daily activities was obtained for up to 12 weeks at a time. Radiographic correction seen was improvement from 14° to 7° on weight bearing radiographs. After five rounds of treatment, no adverse reactions had been observed.

## INTRODUCTION

Hallux varus presents with midline deviation of the hallux at the first metatarsophalangeal (MTP) joint. This joint deformity is most often an unanticipated iatrogenic result from hallux valgus correction surgery, with an incidence between 2 and 15.4% [1]. Other causes can be secondary to trauma, rheumatoid arthritis, infectious agents such as polio, neuromuscular disorders, congenital etiologies or even spontaneous in nature [2, 3].

If left untreated, hallux varus may lead to pain and difficulty with normal daily activities. First MTP joint arthrodesis was traditionally chosen to correct iatrogenic hallux varus deformities, although there exist several soft tissue procedures and non-operative measures such as taping or shoe modification with widened toe boxes [1, 2, 4, 5]. In this report, we present a case of spontaneous hallux varus treated non-operatively with injection of botulinum toxin in the Abductor Hallucis Brevis (AHB).

## CASE REPORT

**Figure 4:**
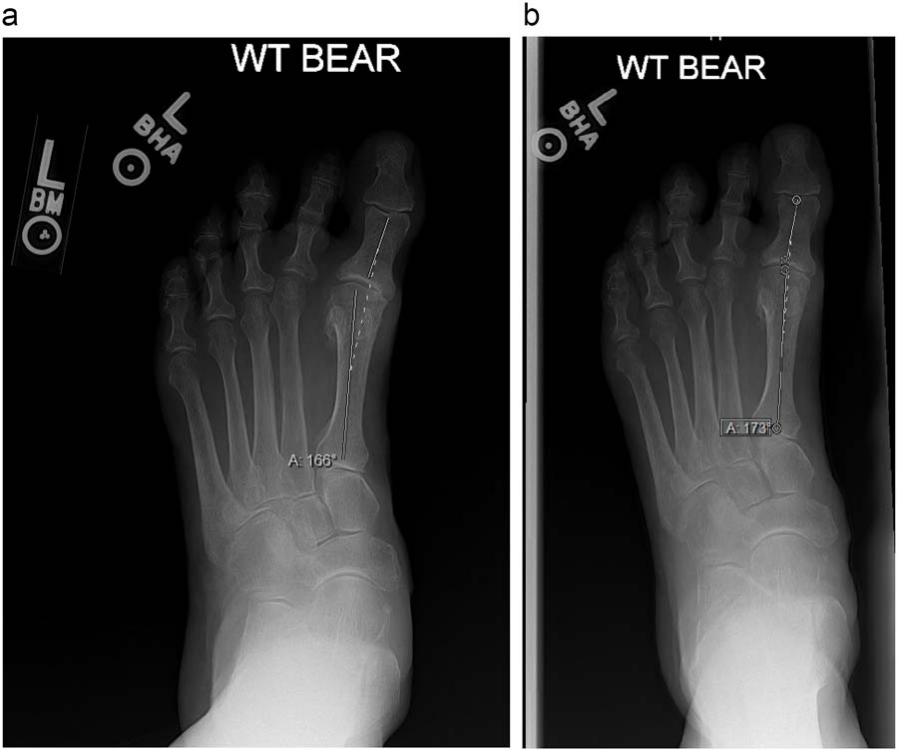
(**a**) and (**b**) are weight-bearing radiographs with angle measurements of the amount of hallux varus at the first MTP joint.

An 84-year-old female presented in clinic with acquired hallux varus of the left foot. She reported that the deformity had been symptomatic for 4 years and that her pain was exacerbated with standing. At the time of presentation, it measured 14° on weight bearing radiographs (Fig. 4A). The deformity was flexible but recurred once the correcting force was removed. The patient had no tendon dysfunction and normal muscle function in the lower extremity. Taping her toe had provided symptomatic relief, but the deformity returned with tape removal. The patient wished to avoid surgery and the use of botulinum toxin for correcting her deformity was proposed by a family member who was a retired orthopedist. After shared decision making, it was agreed that weakening of the AHB with botulinum toxin to reduce the putative deforming force was a reasonable strategy to provide symptomatic relief as well as offer temporary correction to avoid operative treatment. She understood that botulinum toxin injection, if successful, would likely need to be repeated indefinitely.

**Figure 1:**
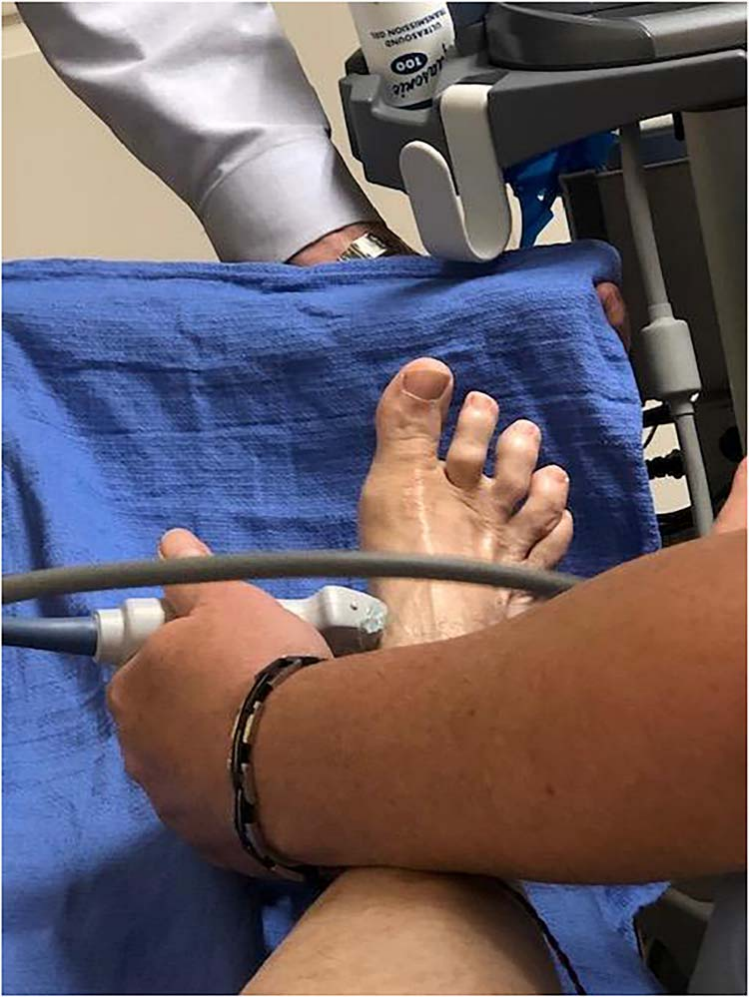
EMG Verification. EMG was used to determine the correct location of the abductor hallux brevis.

**Figure 2:**
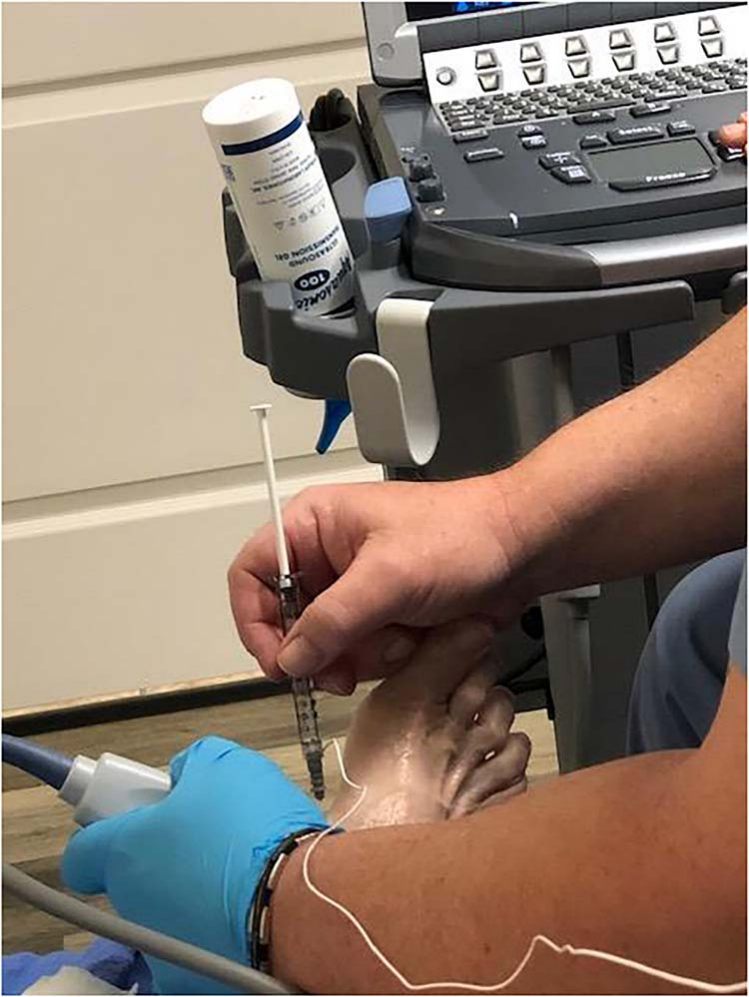
Ultrasound guided botulinum toxin injection. 30-gauge needle was used to inject the Botulinum toxin into the abductor hallucis brevis muscle belly.

**Figure 3:**
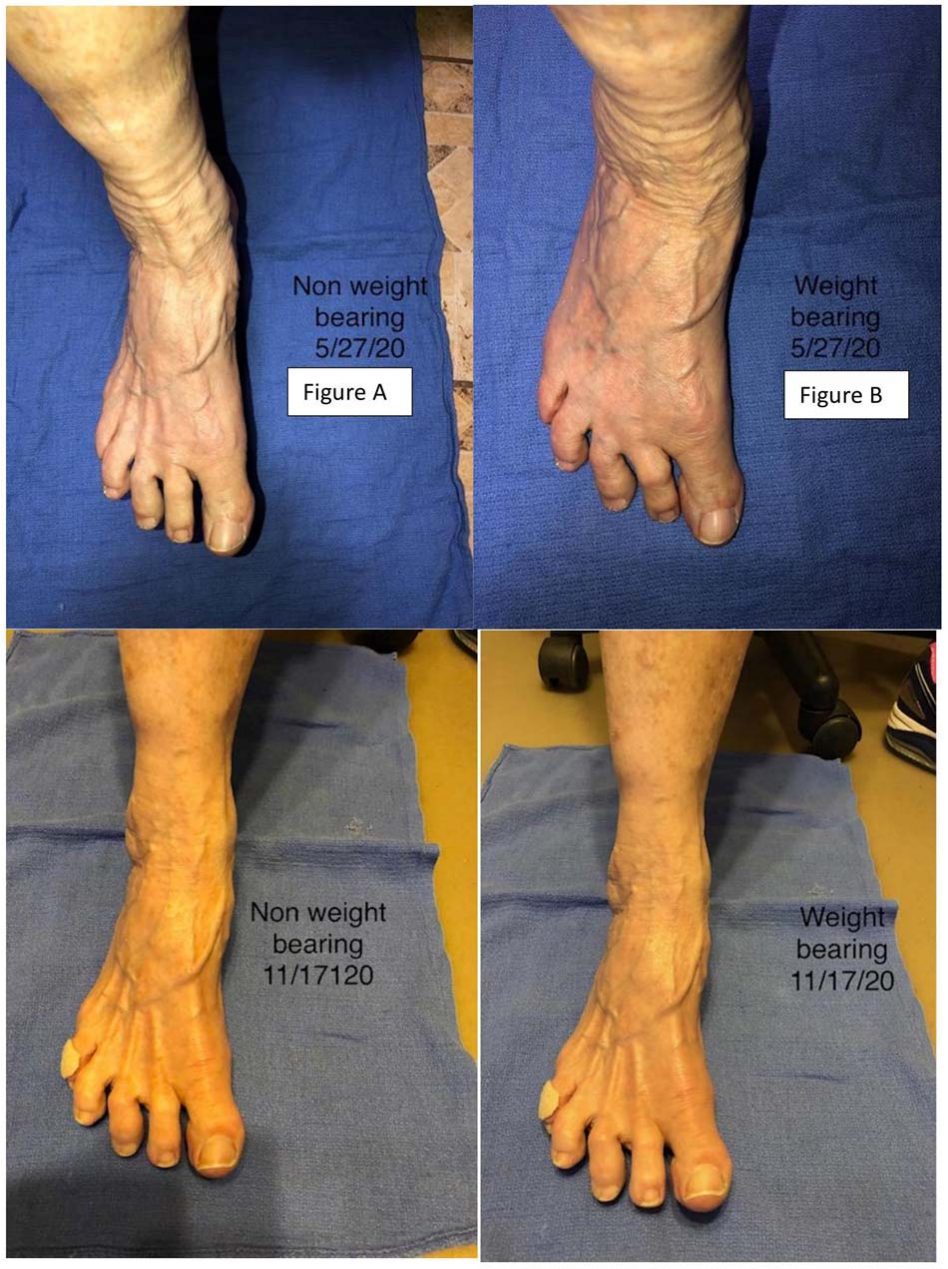
Patient progress images. Images in (**A**) and (**B**) shows the patient’s right foot none weight bearing and bearing weight, respectively, prior to the first injection. (**C**) and (**D**) show the patient at follow-up 2 months after the second injection non-weight bearing and weight bearing, respectively.

After obtaining informed consent, the injection site was cleaned and sterilized. One hundred units of Botulinum toxin were prepared with preservative-free normal saline at a ratio of 100 units/ml using 1 ml syringe. Ultrasound guidance with electromyography (EMG) were used to verify correct location (Fig. 1). A 30-gauge needle was used to inject the Botulinum toxin into the abductor hallucis brevis muscle belly (Fig. 2). No anesthesia was required for the procedure. There was no loss of two-point discrimination or light touch to the forefoot or toes. The procedure was well tolerated and the patient was allowed to walk out of clinic. Figure 3 shows the patient’s foot before and after injection in both non-weight bearing and weight bearing images.

The patient had repeat injections every 3 months, which is customary with botulinum toxin injections as an accepted length in duration of action [6]. Each injection gave symptomatic relief for close to 12 weeks with near total resolution of pain and resumption of activities of daily living. There was no additional adjuvant therapy such as physical therapy, stretching, taping or bracing required. The radiographic correction seen was improvement from 14° to 7° on weight bearing radiographs (Fig. 4B). At her most recent follow-up, the patient reported some pain on the medial aspect of her first toe when in dress shoes but was pain free at the MTP joint when walking barefoot or in walking shoes. Currently, the patient has had five injections with consistency in efficacy and no adverse reactions.

## DISCUSSION

The treatment of hallux varus has made great strides in the last decade. It was originally thought that arthrodesis should be first line due to the perceived propensity of soft tissue procedures to fail in the long-term [7]. However, hallux varus treatment has evolved from first MTP joint arthrodesis to various soft tissue procedures, assuming the deformity is flexible [7]. Gradisek and Weil suggest that arthrodesis only be reserved for treatment-refractory hallux varus with MTP joint damage [7].

Both arthrodesis and tendon transfers have contraindications and complications. Arthropathies, fixed deformities of the MTP joint, deformities with limited motion and excessive resection of the medial eminence are all contraindications for tendon transfers [8]. These patients are often left with no other options besides first MTP arthrodesis. Both arthrodesis and tendon transfers have occasional complications such as hardware failure, joint stiffness, recurrence of deformity or metatarsal head avascular necrosis.

Though limited, there are instances where non-operative management for iatrogenic hallux varus have been suggested [4]. According to Bevernage and Leemrijse, the hallux may be taped into a valgus position if the deformity is recognized quickly enough after surgery [4]. A mild deformity not corrected by taping may be managed by wearing shoes with a wide toe box with padding, to prevent callus formation [4].

In 2013, Rosenberg and Odderson demonstrated the use of botulinum toxin in the AHB to treat a congenital hallux varus deformity. Their electrodiagnostic studies revealed focal dystonia of the AHB [9]. Our report differed in that botulinum toxin in the AHB was used for a spontaneous deformity and further demonstrates an alternative non-operative treatment that may benefit hallux varus patients not desiring surgical intervention or those with contraindications to corrective methods. There has been one reported study investigating the use of botulinum toxin in the symptomatic treatment of hallux valgus that showed promise when compared to placebo [10]. 23 patients were followed over 6 months and improvements in their pain scores and deformity [10]. This was the only study found discussing the use of botulinum toxin in MTP deformity but none were done in the setting of hallux varus.

As hallux varus can leave patients with pain and unsatisfactory cosmetic results, surgical intervention has been the standard treatment for iatrogenic hallux varus. In this case, we demonstrated that use of ultrasound guided botulinum toxin injection into the abductor hallucis brevis muscle belly for hallux varus deformity leads to decreased pain and increased ability to complete daily living activities. This procedure should be considered when dysfunction of the abductor is demonstrated on physical exam or confirmed by EMG. Botulinum toxin injection should be considered as a possible alternative treatment to avoid surgery in these patients.

## Data Availability

Access to the data supporting the case report is restricted due to the Health Insurance Portability and Accountability Act. Deidentified data can be provided by the authors upon request.
